# Comparison of the survival outcomes between retrocolic and antecolic Roux‐en‐Y reconstruction after gastrectomy for gastric cancer

**DOI:** 10.1002/ags3.12779

**Published:** 2024-02-18

**Authors:** Michitaka Honda, Motonari Ri, Takahiro Kinoshita, Hirofumi Kawakubo, Masaki Aizawa, Takeo Bamba, Satoru Matsuda, Hidetaka Kawamura, Mitsumasa Yoshida, Souya Nunobe

**Affiliations:** ^1^ Department of Surgery Southern Tohoku General Hospital Koriyama Japan; ^2^ Department of Minimally Invasive Surgical and Medical Oncology Fukushima Medical University Fukushima Japan; ^3^ Department of Gastroenterological Surgery Cancer Institute Hospital, Japanese Foundation for Cancer Research Tokyo Japan; ^4^ Department of Gastric Surgery National Cancer Center Hospital East Kashiwa Japan; ^5^ Department of Surgery Keio University School of Medicine Tokyo Japan; ^6^ Department of Digestive Surgery Niigata Cancer Center Hospital Niigata Japan

**Keywords:** antecolic route, gastric cancer, retrocolic route, Roux‐en‐Y reconstruction

## Abstract

**Background:**

There are two methods of Roux‐en‐Y (RY) reconstruction after gastrectomy: the antecolic route (ACR) and retrocolic route (RCR). There is no evidence to support that the ACR achieves comparable long‐term survival.

**Methods:**

This was a multi‐center historical cohort study. Patients diagnosed with clinical T3/4a and any N stage who underwent open gastrectomy and R0 resection for gastric adenocarcinoma between January 2006 and December 2012 were enrolled. The primary outcome was the hazard ratio of ACR for overall survival, with adjustment for confounding factors by propensity score matching, and a Cox proportional hazards model.

**Results:**

A total of 1758 eligible patients were identified from the database. After matching, 410 patients in the ACR and RCR groups were included in the final analysis. The adjusted hazard ratio (95% CI) for ACR was 1.148 (0.870–1.492). The five‐year survival rates in the ACR and RCR groups were 74.3% (69.5–78.4) and 77.3% (72.3–81.2), respectively. The short‐term surgical outcomes of the two groups did not differ to a statistically significant extent.

**Conclusion:**

The route used to lift the jejunum in RY reconstruction did not affect the incidence of long‐term survival or postoperative complications. The ACR and RCR are both acceptable options for RY reconstruction during gastric cancer surgery.

## Graphical abstract

The route used to lift the jejunum in Roux‐en‐Y reconstruction after gastrectomy does not affect the long‐term survival. The ACR and RCR are both suitable options for RY reconstruction during gastric cancer surgery.

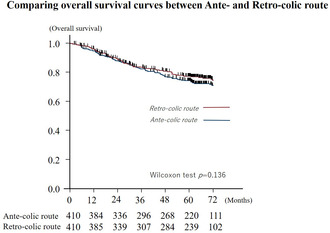

## 1 INTRODUCTION

Roux‐en‐Y (RY) reconstruction after gastrectomy was first reported by the Swiss surgeon César Roux in 1897,^1^, ^2^ and the procedure has been widely used in recent general practice. This reconstruction method involves dissecting the jejunum and pulling it up to anastomose with the esophagus or remnant stomach, and it is a useful reconstruction method in cases of total gastrectomy or subtotal gastrectomy, in which the residual stomach is small. In the case of distal gastrectomy, some previous reports have shown that RY reconstruction shows no significant differences from Billroth I reconstruction in terms of postoperative sequelae, nutritional status, and long‐term curability of gastric cancer.^3^, ^4^, ^5^, ^6^, ^7^, ^8^ RY reconstruction can be performed using two methods: one is to lift the jejunum ventral to the transverse colon (antecolic route, ACR), and the other is to penetrate the mesentery of the transverse colon and lift it (retrocolic route, RCR). Roux et al. reported that the transverse mesocolon was opened and the jejunum was lifted via the RCR.^2^ The ACR has become more widely used in recent years with the spread of laparoscopic surgery in terms of the simplicity of its surgical procedures.

Previous studies have indicated that ACR may be associated with increased postoperative reflux symptoms.^9^ This is because the elevated jejunum is located ventral to the transverse colon and omentum, and may be associated with a risk of bowel obstruction because of contact with the abdominal wall.^10^ Besides, the occurrence of such postoperative disorders might interfere with adjuvant therapy and may also influence long‐term survival outcomes.^11^, ^12^ Regarding the clinical question of whether the ACR or RCR is superior, there is no established consensus. Reports have been based on studies with small sample sizes or questionnaire surveys that have compared short‐term outcomes.^9^, ^10^, ^13^ Furthermore, we have no evidence on the long‐term impact on gastric cancer curability or overall survival outcomes. Surgeons are mainly choosing ACR due to the recent proliferation of minimally invasive surgery, and it is unlikely that any comparative studies between ACR and RCR will be conducted in the future. From a scientific perspective, a retrospective analysis is necessary to confirm that ACR is equivalent to RCR in terms of short‐ and long‐term outcomes.

The present study aimed to compare the long‐term outcomes of the ACR and the RCR using a database constructed from multicenter studies that we have previously reported on the efficacy of omentum‐preserving gastrectomy.^14^ Our hypothesis was that among patients undergoing RY reconstruction after gastrectomy for gastric cancer, the survival outcomes of patients treated using the ACR would not differ from those of patients treated with the RCR.

## 2 PATIENTS AND METHODS

### 2.1 Study design and cohort development

This was a multi‐center historical cohort study. Five Japanese hospitals with a high volume of experience in gastric cancer surgery participated in this study. Consecutive patients who met the following inclusion criteria were enrolled in this study: histologically confirmed gastric adenocarcinoma, diagnosed as clinical T3/4a and any N stage, with R0 resection, including distal or total gastrectomy, between January 2006 and December 2012. The exclusion criteria were laparoscopic surgery, remnant carcinoma in the stomach (after previous gastrectomy), presence of peritoneal dissemination, distant metastasis, other primary malignant disease, history of radiotherapy or chemotherapy, and R1/R2 resection. This study was conducted in accordance with the Declaration of Helsinki and all applicable local laws and regulations. The protocol was approved by the institutional review boards of all participating hospitals. The pathological tumor depth and nodal status were classified according to the eighth edition of the Union for International Union Against Cancer (UICC) classification system.^15^

### 2.2 Surgical procedure and postoperative management

We previously reported our surgical procedures and perioperative management.^14^ Briefly, the standard surgery for locally advanced cancer was open gastrectomy with D2 lymphadenectomy. There are no established criteria for the performance of omentectomy, prophylactic cholecystectomy and splenectomy, or the selection of the reconstructive route (ACR or RCT); therefore, each surgeon makes their own decisions. Adjuvant chemotherapy with S‐1 for was administered 1 year for pathological Stage II/III tumors. All participating hospitals treated gastric cancer patients according to the Japanese guidelines.^16^

### 2.3 Outcomes

The main outcome was the adjusted hazard ratio (HR) of ACR for overall survival (OS). The secondary outcomes included the 5‐year survival rate, recurrence pattern, incidence of postoperative complications according to the Clavien‐Dindo classification,^17^ mortality, and nutritional status at 1 year after surgery, including the percentage of body weight loss, protein concentration, hemoglobin level, and number of lymphocytes.

### 2.4 Propensity score matching and statistical analyses

This was an observational study, and adjustment for confounding factors was essential to properly compare outcomes in both groups. For propensity score matching (PSM), the research team defined measurable parameters potentially associated with the decision of ACR or RCR and the outcome used these factors for the estimation of propensity scores. PSM was performed to adjust for the following covariates: age, sex, body mass index, American Society of Anesthesiologists Physical Status (ASA‐PS), treatment year, clinical TNM factors, site of lesion, tumor size, esophageal invasion, and resection area (total or subtotal gastrectomy, D2 or more lymphadenectomy, prophylactic splenectomy). PSM was conducted by a biostatistician who was blinded to the outcomes. Propensity scores were estimated using a logistic regression model. Optimal matching, in a ratio of 1:1 without replacement, with a caliper of 0.13 standard deviations of the estimated logit, was performed.

Survival outcomes were compared between the ACR and RCR groups using the Kaplan–Meier method and Wilcoxon's test. Hazard ratios (HR) and 95% confidence intervals (CIs) were estimated using an unstratified Cox proportional hazards model for the primary analyses. Descriptive statistics were evaluated for other secondary outcomes, and as necessary, continuous variables were compared using Student's *t*‐test and categorical variables were compared using Fisher's exact test. All statistical tests were two‐sided, and *p* values of <0.05 were considered to indicate statistical significance.

## 3 RESULTS

**FIGURE 1:**
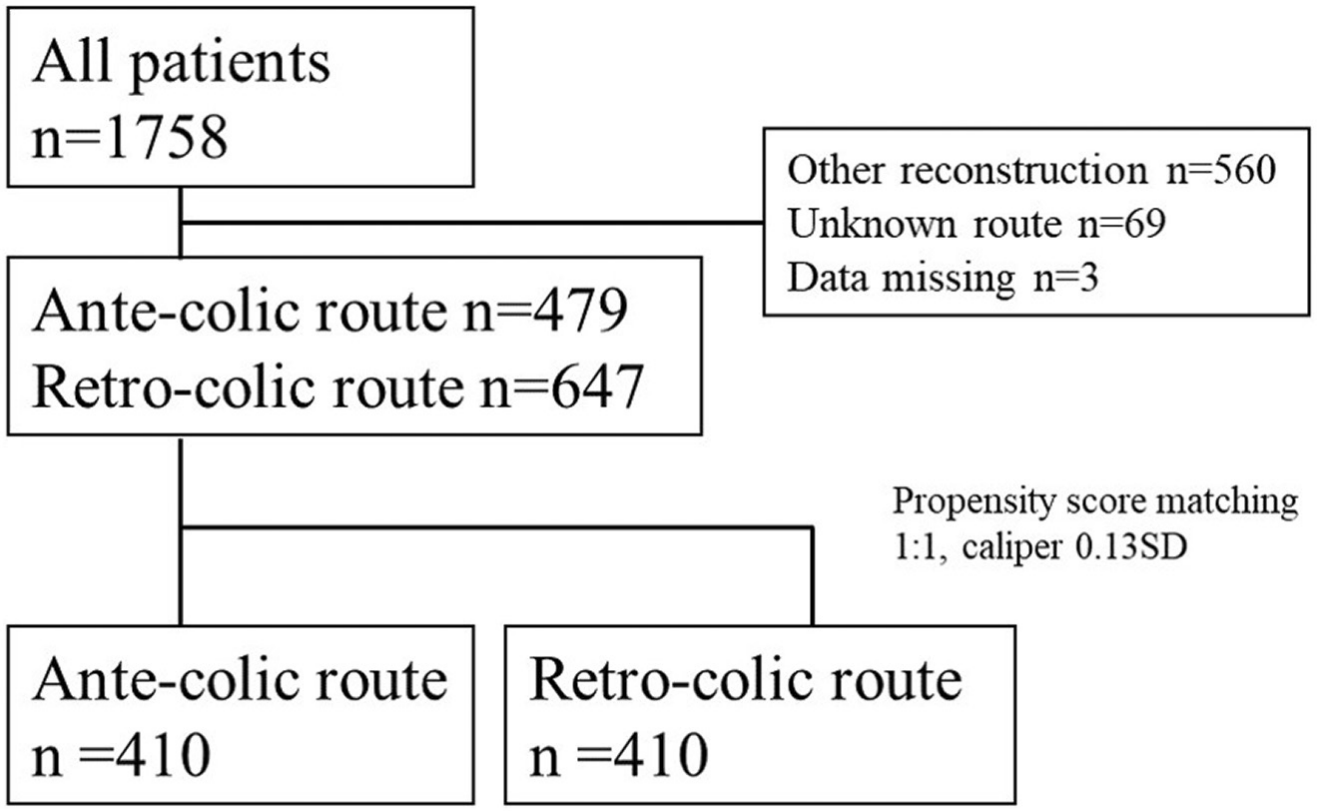
Flow chart of patient enrollment.

Figure 1 shows the flow of patient enrollment. A total of 1758 eligible patients were identified from the database, including 1126 who underwent RY reconstruction: ACR (*n* = 479) and RCR (n647). After matching, 410 patients in the ACR and RCR groups were included in the final analysis. Table 1 shows patient and tumor characteristics.

**TABLE 1:** Patients' characteristics.

	Antecolic route		Retrocolic route		Standardized difference
	*n* = 410	%	*n* = 410	%	
Age					
Median [IQR]	66 [58–74]		66 [58–73]		−3.30
Sex					
Male	288	70.2	290	70.7	−0.11
Female	122	29.8	120	29.3	0.11
Body mass index (kg/m^2^)					
<25	344	83.9	329	80.2	0.29
≥25	66	16.1	81	19.8	−0.30
ASA‐PS					
I	127	31.0	136	33.2	−2.34
II	260	63.4	250	61.0	1.60
III	23	5.61	24	5.85	−0.50
cT					
cT3	132	32.2	131	32.0	0.10
cT4a	261	63.7	265	64.6	−0.05
cT4b	17	4.15	14	3.41	0.20
cN					
cN0	176	42.9	181	44.2	−0.82
cN+	234	57.1	229	55.9	0.82
Tumor size (cm)					
<8	299	72.9	298	72.7	0.11
≥8cm	111	27.1	112	27.3	−0.11
Macroscopic type					
Non‐Type4	390	95.1	390	95.1	0
Type4	20	4.9	20	4.88	0
Histology					
Differentiated	145	35.4	147	35.9	−0.28
Undifferentiated	192	46.8	222	54.2	−3.42
Mixed	70	17.1	37	9.02	5.51
Others	3	0.73	4	0.98	−0.11
Area of resection					
Total gastrectomy	156	38.1	165	40.2	0.32
Distal gastrectomy	254	62.0	245	59.8	−0.32
Extent of lymphadenectomy					
D1–D1+	135	32.9	138	33.7	2.2
D2 or more	275	67.1	272	66.3	−2.2
Resection					
Omentectomy	304	74.2	299	72.9	1.80
Splenectomy	104	25.4	91	22.2	2.23

### 3.1 Adjusted HR and overall survival curves

**FIGURE 2:**
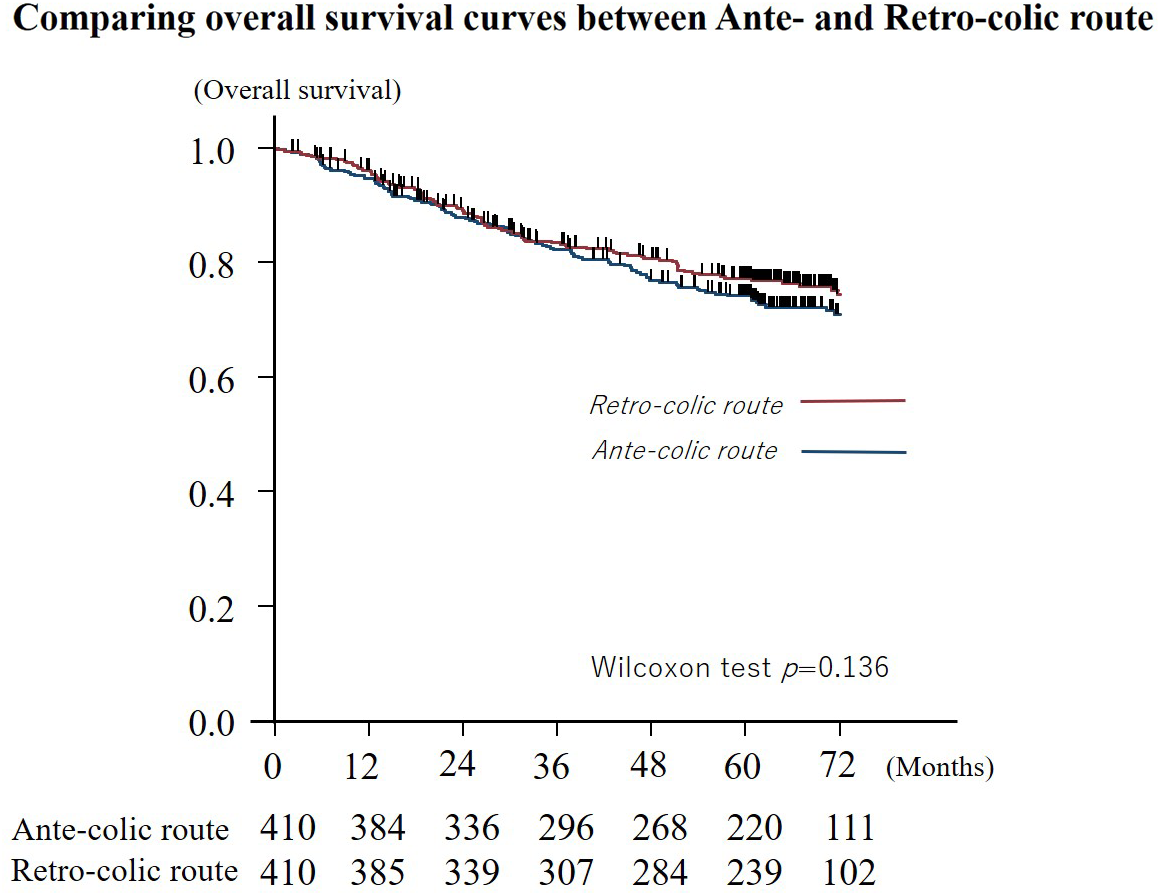
Overall survival curves of the antecolic and retrocolic routes. Overall survival curves were determined using the Kaplan–Meier method. The blue line represents the antecolic route. The red line represents the retrocolic route.

The adjusted HR (95% CI) of the RCR for OS was 1.148 (0.870–1.492). Figure 2 shows the OS rate and the at‐risk population for each group. The 5‐year survival rates in the ACR and RCR groups were 74.3% (69.5–78.4) and 77.3% (72.3–81.2), respectively. The survival outcomes of the two groups did not differ to a statistically significant extent.

### 3.2 Secondary outcomes

Tables 2 and 3 show the surgical outcomes and nutritional status at 1 year after surgery. Table 4 shows the pathological stages and sites of recurrence. The secondary outcomes of the two groups did not differ to a statistically significant extent.

**TABLE 2:** Surgical outcomes.

	Antecolic route		Retrocolic route		*p*‐value
	*n* = 410	%	*n* = 410	%	
Operating time, min (SD)	241.5 (82.9)		241.3 (87.3)		0.975
Blood loss, mL (SD)	305.9 (323.1)		337.8 (325.1)		0.160
Postoperative hospital stay, days	18.4 (17.3)		16.9 (11.9)		0.141
Grade of complication					
II	47	11.5	44	10.7	0.953
III	9	2.2	10	2.4	
≥IV	6	1.5	5	1.2	
Early complications					
Pancreatic fistula	22	5.4	22	5.4	1.000
Intra‐abdominal abscess	12	2.9	11	2.7	
Anastomotic leak	8	2.0	7	1.7	
Late complications					
Adhesive small bowel obstruction	16	3.9	11	2.7	0.783
Internal hernia	5	1.2	5	1.2	
Cholecystitis or cholangitis	5	1.2	5	1.2	
Mortality	1	0.2	1	0.2	1.000

**TABLE 3:** Nutritional indicators 1‐year after surgery.

	Antecolic route		Retrocolic route		*p*‐value
	Mean	SD	Mean	SD	
Body weight loss (%)	12.6	8.84	11.2	10.1	0.189
Serum total protein level (g/dL)	6.801	0.509	6.769	0.51	0.443
Serum albmin level (g/dL)	3.99	0.431	3.98	0.396	0.956
Hemoglobin (g/dL)	11.6	1.56	11.7	1.57	0.291
Lymphocyte (/μL)	820	1064	705	903	0.262

**TABLE 4:** Pathological stage and recurrence.

	Antecolic route		Retrocolic route		*p*‐value
	*n* = 410	%	*n* = 410	%	
Pathological stage					
IA/IB	42	10.2	50	12.2	0.851
IIA/IIB	131	32.0	123	30.0	
IIIA	109	26.6	106	25.9	
IIIB	65	15.9	73	17.8	
IIIC	46	11.2	45	11.0	
IV	17	4.1	13	3.2	
Adjuvant chemotherapy	230	56.1	251	61.2	0.156
Recurrence	110	26.8	98	23.9	0.377
Recurrence site (overlapping)					
Peritoneum	47	11.5	41	10.0	0.573
Liver	25	6.1	19	4.6	0.439
Lymph‐node	24	5.9	20	4.9	0.642
Lung	6	1.5	8	2.0	0.789
Locoregional	6	1.5	6	1.5	1.000
Others	10	2.4	9	2.2	1.000

## 4 DISCUSSION

In the present study, we compared the ACR and RCR in esophagojejunostomy during RY reconstruction after gastrectomy and found two important findings. First, we found no significant differences in oncological outcomes, such as survival or recurrence site, between the two groups. Second, the secondary outcomes of the two groups, including postoperative complications, nutritional status, and the performance of adjuvant chemotherapy.

Few clinical studies have compared the ACR and RCR. Some retrospective studies comparing postoperative complications and sequelae, and some comparative studies of RY bypass in bariatric surgery have been reported.^9^, ^13^, ^18^, ^19^ This study is the first to compare the long‐term oncologic outcomes of patients with gastric cancer. Although our results are associated with the limitations of a historical cohort study, we believe that we succeeded in increasing the comparability of the groups by accumulating a large sample size from multiple centers and performing statistically rigorous PSM to adjust for confounders.

First, whether the route of the jejunum in RY reconstruction is anterior or posterior to the colon does not seem to have a direct effect on gastric cancer recurrence. We also found no significant differences in short‐term outcomes such as operative time, blood loss, or postoperative complications. Although there was concern that ACR might cause stronger tension on the esophagojejunostomy because of the dilatation of the transverse colon or in patients with large omentum volume, the present results showed no difference in the occurrence of anastomotic leakage or stenosis. In addition, because of the contact between the lifted jejunum and abdominal wall in ACR, Korenaga et al. reported a higher risk of small bowel obstruction due to adhesion^10^; however, the results of this study showed no significant difference in the incidence of postoperative bowel obstruction or ileus. Furthermore, in term of long‐term sequela, ACR is associated with an increase in reflux symptoms after eating because the lifted jejunum is diverted ventrally. Hirata et al. reported that in RY reconstruction after distal gastrectomy, the anastomosis is mutated ventrally in the ACR and can cause postoperative esophageal hiatal hernia and reflux esophagitis.^9^ In contrast, Ikeda et al. reported that ACR had a lower degree of reflux symptoms in a survey using a questionnaire, indicating the opposite result.^13^ Although we were unable to investigate reflux symptoms in the current study, we did not find any differences in weight loss or nutritional status, which is analogous to the fact that postoperative sequelae do not differ significantly. ACR and RCR showed no difference in the proportion of patients receiving adjuvant therapy and in survival. We focused on the incidence of sequelae or comorbidities as an essential outcome because it could also affect the completion of postoperative adjuvant therapy and/or oncologic prognosis.^11^, ^12^, ^20^ Based on the results of this study, we believe that the equivalence of ACR compared to RCR, the original method of RY reconstruction, is well established in terms of short‐ or long‐term clinical outcomes.

The present study was associated with some limitations. First, this study enrolled only open surgery cases and did not evaluate laparoscopic or robot‐assisted surgery. It has been suggested that minimally invasive surgery results in less adhesion between the abdominal wall and the intestinal tract, which increases the risk of internal hernia.^21^, ^22^, ^23^ It is possible that in minimally invasive surgery, differences in the route of the jejunum may be more pronounced. In addition, detailed technical issues such as the length of the Y‐limb and anastomosis procedure were not considered. Clinical questions remain regarding these specific surgical techniques to further improve the quality of life of patients who undergo gastrectomy and RY reconstruction.^24^ However, with the current widespread use of minimally invasive surgery,^25^ the application of the ACR has become common in RY reconstruction, and the RCR will be performed less frequently in the future. Therefore, we believe that this study is significant in that it compared the results of the RCR to those of the ACR using retrospective data from open surgery, in which RCR was the standard procedure, and confirmed that the results were comparable in terms of oncologic and nutritional outcomes. Second, patients who underwent reconstruction using the Billroth II method were not included in the current analysis. Few previous studies have reported comparisons of the ante‐ and retro‐colonic routes in the Billroth II method, leaving this issue unresolved.

In conclusion, the short‐ and long‐term outcomes of ACR in RY reconstruction in gastric cancer surgery are similar to RCR. It is recommended that the choice be based on surgeon preference. There is no disadvantage in choosing ACR in laparoscopic or robotic surgery, in which ACR is easier to perform. There is also no disadvantage in performing RCR when ACR is difficult to perform for any reason.

## CONFLICT OF INTEREST STATEMENT

None to disclose.

## FUNDING INFORMATION

No funding was received for this article.

## ETHICS STATEMENT

Approval of the research protocol: All study procedures were conducted in accordance with the ethical standards of the respective committees on human experimentation (institutional and national) and with the Helsinki Declaration of 1964 and later versions. The study was approved by the Institutional Review Board of all participated institutes.

Informed Consent: N/A.

Registry and the Registration No. of the study/trial: N/A.

Animal Studies: N/A.
